# Enablers of students’ health-promoting lifestyle: evidence from PLS-SEM and fsQCA

**DOI:** 10.3389/fpubh.2025.1667920

**Published:** 2025-12-22

**Authors:** Lanxing Zhang, Fuxue Zhao

**Affiliations:** Hubei Collaborative Innovation Center for Scientific Sports and Health Promotion, Wuhan Sports University, Wuhan, China

**Keywords:** adolescence health-promoting lifestyle, emotion regulation, empathy, self-control, body image

## Abstract

Adolescent health-promoting lifestyles, which are of global concern, significantly influence personal well-being. Previous research has highlighted the close connection between emotional competence and adolescent health behaviors and lifestyles. However, there is limited understanding of the underlying mechanisms between specific dimensions of emotional competence and adolescent health-promoting lifestyles. The study included 1,163 adolescents (female = 52.5%, age mean = 13.29, SD = 0.8) structural equation model (SEM) and fuzzy set qualitative comparative analysis (fsQCA) were used to investigate the interrelationships among adolescents’ emotional competence, body image, social media use, and the promotion of a healthy lifestyle. According to the SEM results, emotional regulation, self-control, and empathy can all account for body image and subsequently affect adolescent health-promoting lifestyles. The results of fsQCA indicate that there are multiple configurations among emotional regulation, empathy, self-control, body appreciation, and social media use that lead to a high level of health-promoting lifestyle for adolescents. Furthermore, social media use was found to positively moderate the relationship between body appreciation and a health-promoting lifestyle. These findings can assist families and schools in formulating corresponding strategies during adolescent growth, help adolescents enhance their emotional competence, and establish a positive body image to promote the formation of a health-promoting lifestyle.

## 1 Introduction

**Figure 1:**
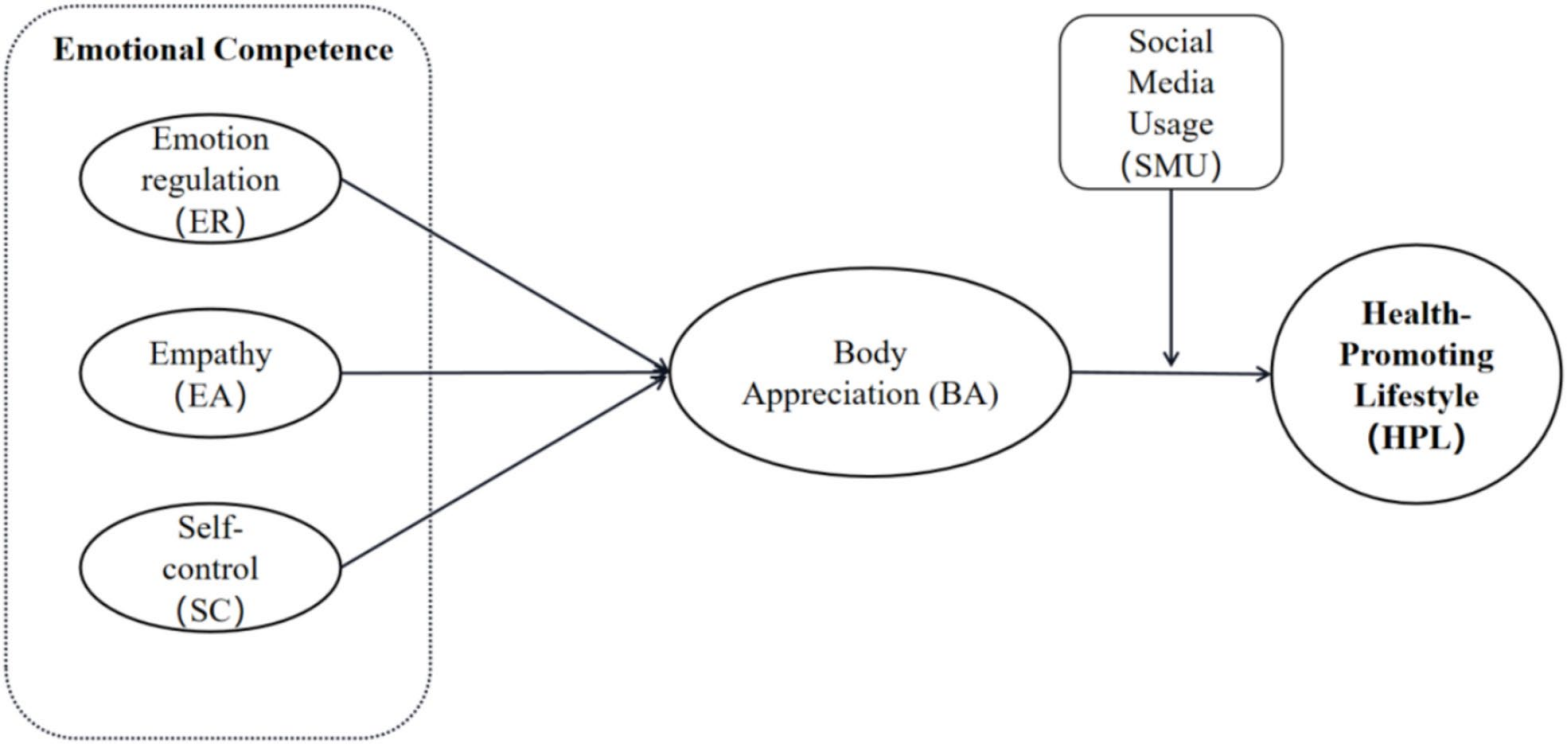
Linear influence model of health-promoting lifestyle.

The healthy development of adolescents has invariably been a crucial topic in social development. Given the escalating rates of adolescent obesity, myopia, sedentary behavior, and unhealthy interpersonal communication (1), along with shifts in lifestyle and psychological well-being in the post-COVID-19 era, strengthening the management of adolescents’ emotional competence and facilitating their establishment of a health-promoting lifestyle (HPL) among this population. These have become key global concerns and essential components of social welfare. A health-promoting lifestyle generally encompasses behaviors related to nutrition, health responsibility, and physical activity (2). It represents a set of health-oriented behavioral patterns that individuals choose based on their life circumstances (3), aimed at realizing health potential. Previous research has largely focused on demographic variables, such as gender (4), race (5), and occupation (6), or external perspectives like social support (7) and public health policies (8). However, limited studies examined how adolescents themselves mobilize internal resources to develop such lifestyles. According to the theory of planned behavior (9) and the health belief model (10), the shift toward healthy behavioral lifestyle originates from individuals’ conscious actions to take responsibility for their own health. Adolescence, as a critical period for identity formation, with body appreciate serving as its core component. Moreover, social media has become a primary platform for social comparison and body image perception (11, 12). Therefore, this study focuses on the roles of body appreciate and social media use, in conjunction with emotional competence, in shaping adolescents’ health-promoting lifestyles. By investigating the relationships among these factors, this research aims to provide a theoretical foundation and practical strategies to support adolescents’ physical and mental health. Enhancing emotional competence, fostering positive body image, and promoting rational social media use can effectively guide adolescents toward adopting sustainable healthy lifestyles (See Figure 1).

### 1.1 Emotional competence and health-promoting lifestyle

Emotional competence (EC) is a multidimensional psychological construct (13), encompasses core capacities including emotion regulation (ER), self-control (SC), and empathy (EA) (14, 15). It serves as a function for individuals to establish, maintain, and alter connections with the external world (16, 17). The efficacy of emotional competence in behavior change has been thoroughly verified (16, 18, 19). Research indicates that non-cognitive skills are correlated with future health outcomes. Specifically, children with more advanced emotional competence in early development are at a lower risk of substance use and mental health problems in adolescence and adulthood (20). This suggests that well-developed emotional competence helps adolescents effectively manage emotional states when facing life stressors and challenges (21), thereby mitigating the adverse effects of health anxiety and supporting the maintenance of a positive, health-promoting lifestyle. Although EC operates as a holistic construct, examining its distinct dimensions offers valuable insights into the specific mechanisms through which it influences health this study, therefore, investigates the unique contributions of these constituents of ER, SC, and EA to adolescents’ health-promoting lifestyles, recognizing that both their individual effects and interrelationships may reveal distinct pathways to the formation of health behaviors.

Emotion regulation (ER) refers to the process through which individuals monitor, evaluate, and modify their emotional responses, thereby influencing the occurrence, experience, and expression of emotions (22). Research has indicated that there exists a correlative relationship between emotion regulation and the maintenance of a healthy lifestyle by breast cancer survivors (23). This furnishes a research foundation for exploring the connection between emotion regulation of adolescents and health-promoting lifestyles. Diet is a prominent manifestation of the link between emotion regulation ability and a healthy lifestyle. Individuals with inadequate ER capacity tend to experience greater difficulties in managing their diet under high pressure and are prone to develop unhealthy eating habits (24). A study of 115 obese adolescents demonstrated that ER strategies significantly positively affect emotional eating and support weight loss efforts (25). Conversely, impairments in ER are associated with maladaptive stress responses (26). Individuals with poor emotion regulation frequently employ maladaptive behaviors to escape or reduce their emotions (27), such as bulimia nervosa (28), sleep disorders (29), tic disorders (30), all of which pose direct threats to adolescent health. Existing studies have affirmed that effective ER supports behavioral adaptation in children and adolescents (26). For instance, a study of 1,221 adolescents aged 10 to 17 reported that ER helps reduce procrastination (31). Moreover, ER strategies are widely incorporated into interventions targeting maladaptive social behaviors and behavioral disorders (32, 33). This suggests that individuals with well-developed ER are more likely to manage their behaviors and decisions when confronted with challenges, avoid health-compromising choices driven by emotional fluctuations, and adopt healthy lifestyle. Thus, the first hypothesis is put forward.

*H1a*: Emotion regulation positively predicts adolescents' health-promoting lifestyles.

Empathy (EA) refers to an individual’s capacity to recognize and understand others’ emotional states and to generate congruent emotional and behavioral responses (34). Substantial evidence suggests that promotes altruistic in adolescents, and plays a crucial role in their interpersonal functioning (35). This can be construed as individuals with greater EA are more capable of sharing others’ emotions (36) and adopting others’ perspectives, which in turn facilitates social support (37), fosters positive peer relationships, and improves interpersonal outcomes (38). This can also elucidate the formation process of healthy behaviors in adolescents. Those with a high levels of EA may be moved by others’ experiences, and encourage kinder self-attitudes (39). As a result, they possess more psychological resources for self-care, enhance their management of healthy behaviors, and gradually form a health-promoting lifestyle (40). This view has been supported by research during the COVID-19 pandemic, which found that EA was positively associated with physical distance and wearing masks during the pandemic. Higher EA encourages individuals to comply with relevant requirements beneficial to their own and others’ health (41). Accordingly, the following hypothesis is put forward:

*H1b*: Empathy is positively associated with adolescents' health-promoting lifestyles.

Self-control (SC) refers to an individual’s capacity to refrain from engaging in short-term behaviors that are instantly gratifying but disadvantageous in the long run (42). Adolescence represents a stage of rapid development of self-control. Adolescents with higher levels of SC are more capable of controlling their thoughts and suppressing impulses (43), which contributes to improved physical and mental health (43) and academic achievement (44). In the domain of health promotion, empirical studies have shown that SC is positively associated with almost all forms of behaviors that conducive to successful and healthy living, such as maintaining a balanced diet and engaging in physical activities (45). Conversely, it is negatively correlated with health-compromising behaviors, like non-suicidal self-injury (46), alcohol consumption (47), and e-cigarette use (48). Additionally, SC is closely linked to the spiritual growth of adolescents. Those with strong SC can more effectively manage their emotions and mitigate the impact of negative emotional such as excessive anxiety and depression (49). They tend to adopt positive coping strategies to maintain a good psychological state, in positive ways, like exercising, reading, and communicating with others to maintain a good psychological state.

A reasonable conjecture is put forward that self-control, as a positive psychological force capable of directly influencing behavior, helps adolescents establish defensive against unhealthy environments and behaviors. Specifically, a high level of SC prompts adolescents to pursue long-term objectives of a healthy life and adhere to exercise, a nutritious diet, and a regular lifestyle, gradually forming a health-promoting lifestyle. Accordingly, the following hypothesis is proposed:

*H1c*: Self-control is positively associated with adolescents' health-promoting lifestyles.

### 1.2 Emotional competence and body appreciation

Body appreciation (BA) was advanced by Paul Schilder (50), referring an individual’s perception of their own physical appearance, encompassing the physiological functional characteristics of the body and the individual’s attitude towards these traits. Pokorny (51) describes BA as a collection of fantasies and meanings associated with the body and its functions. Other scholars have further characterized it as a mental representation of one’s own body (52). In summary, BA constitutes a subjective, psychologically grounded perception of one’s physical traits, which forms a basis for examining its relationship with emotional competence. Notably, during early adolescence, the physical self becomes a central element of self-concept, as adolescents undergo rapid bodily changes and increased self-awareness. At this stage, their body appreciation is particularly susceptible to external influences (53). Despite its relevance, few empirical studies have connected emotional competence with BA, particularly in adolescent populations. Specifically, it helps individuals develop a more positive and accurate perception of their bodies adaptive emotion regulation strategies, thereby achieving the purpose of promoting individual physical and mental health (54, 55). These findings offer new perspectives into the relationship between ER and BA. Empirical studies have shown that individuals with emotion regulation difficulties tend to exhibit heightened body-related concerns and are more likely to develop negative body appreciation (56). This suggests that effective ER may help individuals maintain a positive outlook toward their bodies. When faced with external criticism or personal dissatisfaction regarding their appearance, they can mitigate such negative emotions by means of positive self-talk, distraction and other approaches, thereby maintaining a relatively stable and positive body appreciation. Accordingly, the following hypothesis is put forward.

*H2a*: Emotion regulation positively predicts adolescent body appreciation.

Empathy, a multidimensional capacity to understand and response to others’ emotions, is positively associated with BA and its positive influences. Individuals with a high levels of EA are more likely to comprehend diverse views and feelings regarding the BA, and they demonstrate greater acceptance and positive perception of BA that deviate from prevailing social standards (57). For instance, one study has found that consumers with a relatively high level of empathy are more capable of understanding and accepting plus-size models appearing in clothing brand campaigns (58). This suggests that it can be observed that individuals with a high level of empathy are better able to experience and embrace the existence of body diversity, which in turn helps them develop a more objective and inclusive BA and reduces their adherence to narrow beauty ideals. The following hypothesis is put forward:

*H2b*: Empathy positively predicts adolescent body appreciation.

Self-control is considered essential for individuals to navigate and adapt effectively to their surrounding environment (59). Research suggests that higher levels of SC foster more positive emotions, which aid in coping with external challenges (60). At the same time, SC is subject to situational influences. When adolescents face adverse circumstances, SC enables them to resist temptations or impulses in pursuit of long-term goals (42). Compared to individuals with low SC, those with greater SC are better able to manage their thoughts and regulate emotional responses (42).

It follows that adolescents with strong SC may be better equipped to handle external evaluations related to their bodies. They can maintain a rational and objective attitude and uphold a stable and positive BA. Accordingly, the following hypotheses are proposed.

*H2c*: Self-control positively predicts adolescent body appreciation.

### 1.3 Body appreciation and health-promoting lifestyle

During adolescence, BA forms a core element of self-concept, particularly among junior high school students undergoing rapid physical and psychological development. At this stage, adolescents’ perceptions of their own bodies are highly susceptible to external influences. BA derived from one’s own body shape, are closely associated with lifestyle. For instance, Unhealthy BA is frequently accompanied by unwholesome eating behaviors. Reducing calorie intake is a relatively common method, mainly manifested as consuming low-calorie foods or engaging in intermittent fasting (61). However, sustained insufficient calorie intake may lower metabolic rate and heighten appetite, potentially triggering episodes of binge eating and leading to more rapid weight regain (62). In some cases, dieting and vomiting behaviors may occur (63), plunging into a vicious cycle of unhealthy eating. Whereas early research emphasized negative BA. Recent scholarship has gradually shifted towards the relationship between positive body appreciation and personal behaviors. Positive body appreciation can act as a protective mechanism (64). Individuals with a positive body appreciation are more resistant to received negative information (such as “ideal thinness”) (65) and will exhibit fewer unhealthy eating behaviors and generate more healthy ones (66).

Beyond eating behavior, body appreciation also serves as a motivator for physical activity. Weight dissatisfaction and weight-control behaviors do not necessarily reflect actual obesity (67). Many commonly employed weight-control strategies—such as drinking more water, increasing fruit consumption, reducing sweets, and engaging in regular exercise—are also core components of a health-promoting lifestyle. Furnham and Greaves observed that exercise participation is often influenced by body-related attitudes, suggesting that many individuals exercise to maintain or alter body shape, enhance appearance, and ultimately promote health (68). This view is supported by studies noting significant correlations among weight concerns, body dissatisfaction, and exercise engagement in adolescent females, with body appreciation serving as a primary motivator for maintaining a healthy diet and active lifestyle (69). Similarly, another study reported that more positive body appreciation is associated with higher exercise frequency (70). Notably, the influence of body appreciation on health-promoting lifestyles is not confined to females. Among males, the pursuit of a healthy appearance closely linked to exercise behavior (71), often characterized by muscularity, low body fat, and a V-shaped torso (72).

In summary, while emotional competence and health-promoting lifestyles represent broad, multidimensional constructs, BA provides a tangible behavioral and psychological pathway through which macro-level emotional capacities translate into concrete lifestyle outcomes, accordingly, the following hypotheses are put forward.

*H3*: Body appreciation positively predicts adolescents' health-promoting lifestyle.

Emotional competence (including ER, EA, and SC) provides essential psychological resources for constructing a positive BA. Specifically, ER allows individuals to effectively manage BA related anxiety and stress (73), preventing their internalization into negative self-perceptions (74). EA fosters a kind and non-judgmental acceptance of bodily limitations and imperfections (75). Meanwhile, SC empowers individuals to resist impulsive, unhealthy body management practices, such as extreme dieting, in favor of sustained health-promoting behaviors (76). The synergy of these emotional resources cultivates a robust sense of BA. This internalized positive body image, in turn, fundamentally shifts the motivation for health behaviors from an extrinsic, individuals become more inclined to attune to their body’s authentic needs, opting for balanced nutrition, engaging in physical activities that celebrate bodily vitality and function, and proactively assuming responsibility for their health and well-being (77). Thus, BA acts as a pivotal psychological process that effectively bridges emotional competence with a health-promoting lifestyle, elucidating the intrinsic pathway through which positive emotional skills foster lasting, self-determined health behaviors via the shaping of a positive BA. Based on the aforementioned proposed relationships between emotional competence dimensions and body appreciation (H2a-c), and between body appreciation and health-promoting lifestyle (H3). Thus, the following mediating hypotheses are proposed:

*H4a*: Body appreciation plays a mediating role between emotion regulation and adolescents' health-promoting lifestyle.

*H4b*: Body appreciation plays a mediating role between empathy and adolescents' health-promoting lifestyle.

*H4c*: Body appreciation plays a mediating role between self-control and adolescents' health-promoting lifestyle.

### 1.4 Social media usage

Social media defined as Internet-based social interaction service platform, and adolescents are more likely to utilize social media than groups in other age brackets (78). A nationwide survey in China revealed that 61.8 and 65.1% of adolescents use short-video platforms (such as TikTok, BiliBili, etc.), and 72.1 and 77.0% of students use social media (Weibo, Facebook, etc.) (79). Given that adolescence represents a critical period for the formation of self-concept, particularly BA, social media exerts a profound influence on how adolescents perceive and evaluate their bodies. Specifically, for adolescents with higher levels of SMU, the positive pathway from BA to HPL may be strengthened. Frequent interaction with these platforms increases exposure to body-related information, including health, fitness, and body-positive content (80). When adolescents with high BA encounters such content, it can serve to repeatedly validate, contextualize, and reinforce their internal positive body image. This process may further strengthen their conviction and provide concrete examples or social support for translating their positive BA into tangible HPL, such as choosing nutritious foods or engaging in enjoyable physical activity (81). This perspective aligns with existing evidence (82). Therefore, SMU is theorized not merely as a source of risk or benefit, but as a powerful contextual lens. It moderates the efficacy with which body appreciation is converted into a health-promoting lifestyle, with higher usage potentially intensifying this psychological-behavioral link by providing a pervasive environment that constantly engages with and reinforces body-related attitudes and behaviors. The following hypotheses are put forward.

*H5a*: The mediating effect of body appreciation between emotion regulation and adolescents' health-promoting lifestyle is moderated by social media use.

*H5b*: The mediating effect of body appreciation between empathy and adolescents' health-promoting lifestyle is moderated by social media use.

*H5c*: The mediating effect of body appreciation between self-control and adolescents' health-promoting lifestyle is moderated by social media use.

## 2 Methods

### 2.1 Data collection and procedures

This survey was conducted in May 2024 using a convenience sampling approach across 15 junior high schools in Shenzhen, China through convenient sampling. Data collection was primarily carried out through paper-based questionnaires. The researchers elucidated the concepts and purposes of the study to the students who were interested in participating. After the students consented and signed the informed consent form, they received a paper questionnaire and completed it. This study has obtained the consent of all participants and their guardians to participate. This study was conducted in accordance with the ethical guidelines of them Declaration of Helsinki and approved by the Institutional Review Board (IRB) of Wuhan Sports University (Ethics Approval No. 2024118 Approval Date: 2024.02.17). A total of 1,300 questionnaires were distributed, and 1,176 were returned. After excluding 13 invalid responses, 1,163 valid questionnaires were retained, resulting in an effective response rate of 89.5%. Of the total, 553 were male (47.5%), and 609 were female (52.5%). At the age of 11 accounts for 0.2%. At the age of 12 accounts for 15.7%. At the age of 13 accounts for 45.6%. At the age of 14 accounts for 32.3%. At the age of 15 accounts for 6.2%.

This study employed a hybrid analytical approach combining partial least squares structural equation modeling (PLS-SEM) and fuzzy-set qualitative comparative analysis (fsQCA). PLS-SEM is utilized to calculate the path coefficients and significance levels between latent variables and reduce the error in the model (83). Given the interdependencies among factors influencing adolescents’ health-promoting lifestyle, fsQCA is employed to analyze the relationship between the combinations of various dimensional factors and the results and explore the configuration paths of adolescents’ health-promoting lifestyle.

### 2.2 Instrumentation

#### 2.2.1 Emotional competence scale (ECS)

The emotional competence scale in the *Chinese Positive Psychological Assessment Manual* (84) was adopted. It includes three dimensions: emotion regulation, empathy, and self-control, with a total of 9 items. A 6-point Likert scale was used (1 for “strongly disagree” and 6 for “strongly agree”). The total score is 54. The higher the score, the stronger the emotional competence of adolescents. The Cronbach’s *α* for this instrumentation was 0.781 ~ 0.832.

#### 2.2.2 Health-Promoting Lifestyle Profile (HPLPII-R)

The Chinese revised version (HPLPII-R) of the Health-Promoting Lifestyle Profile II developed by Walker et al. (85) was adopted. This scale was revised by Cao Wenjun et al. (86). It includes six dimensions: nutrition (N), health responsibility (HR), physical activity (PA), stress management (SM), interpersonal relationship (IR), and spiritual growth (SG). A Likert 4-point scale is used (1 for “never” and 4 for “always”). The higher the score, the more willing the individual is to adopt a health-promoting lifestyle. The Cronbach’s α for this instrumentation was 0.853 ~ 0.937.

#### 2.2.3 Body Appreciation Scale-2 (BAS-2)

The Body Appreciation Scale-2 revised by Tylka and Wood Barcalow (87) was adopted. It includes ten items covering the degree of appreciation for the body, the degree of broadly defining beauty, the degree of acceptance of the body, and the degree of influence of internal positivity on external behavior. A Likert five-point scale is used (1 indicates “completely in line” and 5 indicates “completely inconsistent”). The higher the score, the higher the degree of appreciation and acceptance of one’s own body. The Cronbach’s α for this instrumentation was 0.941.

#### 2.2.4 Social media usage intensity scale (SMUIS)

The intensity questionnaire of social networking site use revised by Sun Xiaojun et al. (88) based on the one compiled by Ellison, Steinfeld (89) was adopted. There are a total of six items. A Likert 5-point scale is used (1 indicates “very inconsistent” and 5 indicates “very consistent”). It measures the intensity of emotional connection between individuals and social networking sites and the degree to which social networking sites are integrated into individuals’ lives. After standardizing the scores of all items and adding them up, the higher the score, the greater the intensity of social media use. The Cronbach’s α for this instrumentation was 0.860.

### 2.3 Rationale for using PLS-SEM and fsQCA

This study adopts an integrated strategy combining partial least squares structural equation modeling (PLS-SEM) and fuzzy-set qualitative comparative analysis (fsQCA) to comprehensively and deeply explore the complex relationships among adolescents’ emotional competence, body appreciation, social media use, and health-promoting lifestyles. PLS-SEM is applied to validate the theoretical model proposed in this study, examine the net effects among constructs, and test the significance of hypotheses H1–H5. This method is suitable for assessing the linear relationships among various variables in the model and their overall explanatory power. However, adolescent health behaviors often emerge from the confluence of multiple factors and can be realized through distinct causal pathways, meaning several different combinations of variables may lead to the same outcome. To thoroughly uncover these underlying complex causal mechanisms, and given the comprehensive and complex nature of the impact of emotional competence on adolescents’ healthy lifestyles as well as the inherent complexity of healthy lifestyles themselves, an asymmetric research approach is required to investigate the intricate influence of adolescents’ emotional competence on their healthy lifestyles. By integrating PLS-SEM and fsQCA, this study aims to provide both generalizable insights into variable relationships and a deeper understanding of the causal complexities underlying adolescents’ health behavior patterns.

It is important to emphasize the complementary nature of the PLS-SEM and fsQCA in this study. PLS-SEM examines the net effect of individual variables while controlling for others, testing specific hypotheses derived from theory. In contrast, fsQCA adopts a configurational perspective to identify multiple, equifinal combinations of conditions that are sufficient for the outcome. Accordingly, in all PLS-SEM analyses (including mediation and moderated mediation), the effects of other emotional competence dimensions and demographic variables were controlled for to isolate unique relationships. The fsQCA results, however, illustrate how these factors may combine in different, and often substitutable ways within real-world contexts, thereby offering a nuanced understanding of causal complexity beyond net effects.

## 3 Results

### 3.1 Partial least squares structural equation modelling (PLS-SEM)

#### 3.1.1 Common method bias test

In this study, the Harman single-factor method was employed to examine common method bias. All items encompassed in the research scale were incorporated into factor analysis. Through the utilization of principal component analysis, a total of 11 common factors with eigenvalues greater than 1 were obtained, and the cumulative variance explained was 65.291%. Among these, the variance contribution rate of the first unrotated factor was 31.261%, which is less than 40%, suggesting that there is no significant common method bias in this study (90).

#### 3.1.2 Multicollinearity test

Multicollinearity was examined through variance inflation factor (VIF) and tolerance. A multiple linear regression analysis model was constructed via SPSS. The examination results indicate that the VIF and tolerance values of all predictor variables are below the recommended thresholds. Consequently, it can be concluded that there is no significant multicollinearity issue among the independent variables in this study. The maximum value of the Pearson correlation coefficient between variables is 0.598, which is lower than the standard value of 0.7, suggesting that there is no severe multicollinearity problem in this research (91) (see Table 1).

**Table 1:** Descriptive analysis and bivariate correlations among key variables.

Variable	M	SD	01	02	03	04	05	06
01 ER	4.895	0.919	1					
02 EA	4.854	1.030	0.471**	1				
03 SC	4.900	0.970	0.528**	0.437**	1			
03 BA	3.945	0.904	0.583**	0.572**	0.591**	1		
04 HPL	3.021	0.554	0.516**	0.458**	0.486**	0.598**	1	
05 SMU	3.293	1.096	0.105**	0.145**	0.062*	0.128**	0.163**	1

**p < 0.01.

#### 3.1.3 Reliability and validity test

To validate the reliability and validity of the questionnaire data, Cronbach’s alpha coefficient and composite reliability (CR) are employed to assess reliability. The Cronbach’s alpha coefficient are 0.781 ~ 0.941, and CR value of the data for each variable item are 0.873 ~ 0.956, all above 0.7, suggesting that each scale possesses good reliability. The average variance extracted (AVE) of all variables is above 0.5, demonstrating good convergent validity.

The HTMT statistic and Fornell-Larcker criterion are utilized to examine the discriminant validity among variables. The HTMT ratio between each dimension does not exceed 0.85, and the square root of the average variance extracted (AVE) of each variable is greater than the correlation coefficient between each variable, indicating excellent discriminant validity (see Table 2).

**Table 2:** Reliability and validity analysis results.

Construct	Cronbach’s	CR		AVE
	alpha	CR (rho_c)	CR (rho_a)	
ER	0.832	0.899	0.832	0.749
EA	0.849	0.909	0.853	0.769
SC	0.781	0.873	0.781	0.696
BA	0.941	0.95	0.941	0.654
IR	0.854	0.896	0.855	0.632
HR	0.937	0.946	0.938	0.614
SM	0.82	0.874	0.822	0.583
N	0.853	0.891	0.854	0.577
PA	0.886	0.909	0.887	0.557
SG	0.896	0.923	0.900	0.708
SMU	0.860	0.895	0.861	0.588
HPL	0.913	0.931	0.956	0.692

#### 3.1.4 Structural model assessment

As indicated in Table 3, ER exerts a significant positive influence on HPL (*p* < 0.001, *β* = 0.190), thus H1a is validated. EA has a significant positive impact on HPL (*p* < 0.01, *β* = 0.091), and consequently H1b is established. SC shows a significant positive effect on HPL (*p* < 0.001, *β* = 0.131), and therefore H1c is affirmed.

**Table 3:** Results of structural model assessment.

Pathways	Bootstrap	S. E	t	*p*	95%CI	
	β				Lower	Upper
ER → HPL	0.190	0.033	5.772	<0.001	0.126	0.255
EA → HPL	0.091	0.030	3.014	0.003	0.031	0.148
SC → HPL	0.131	0.036	3.684	<0.001	0.060	0.200
ER → BA	0.273	0.032	8.593	<0.001	0.208	0.333
EA → BA	0.306	0.035	8.732	<0.001	0.236	0.374
SC → BA	0.314	0.035	9.080	<0.001	0.248	0.384
BA→HPL	0.379	0.038	9.944	<0.001	0.305	0.454
SMU → HPL	0.084	0.024	3.519	<0.001	0.038	0.132
SMUx BA→HPL	0.147	0.029	4.998	<0.001	0.090	0.205

Regarding the relationships with BA, the following results were obtained. As presented in Table 3, ER shows a significant positive impact on BA (*p* < 0.001, *β* = 0.273), thereby supporting H2a. Similarly, EA exhibits a significant positive influence on BA (*p* < 0.001, *β* = 0.306), confirming H2b. SC also has a significant positive effect on BA (*p* < 0.001, *β* = 0.314), validating H2c. Moreover, BA has a significant positive impact on HPL (*p* < 0.001, *β* = 0.379), establishing H3.

#### 3.1.5 Mediation analysis

Table 4 shows the rest results of the mediation effect of BA on ER and HPL, the standardized mediating effect 0.104, with a confidence interval of [0.073, 0.137], which does not contain 0, indicating there is a significant mediating effect of BA between ER and HPL, and thus H4a is corroborated.

**Table 4:** Results of mediating effect test.

Effect	Bootstrap β	S. E	95%CI	
			Lower	Upper
Total effect	0.294	0.031	0.235	0.357
Direct effect	0.190	0.033	0.126	0.255
ER → BA→HPL	0.104	0.016	0.073	0.137
Total effect	0.206	0.031	0.145	0.265
Direct effect	0.091	0.030	0.031	0.148
EA → BA→HPL	0.116	0.017	0.084	0.151
Total effect	0.250	0.033	0.184	0.314
Direct effect	0.131	0.036	0.060	0.200
SC → BA→HPL	0.119	0.018	0.086	0.158

Similarly, the standardized mediating effect of BA between EA and HPL is 0.116, with a confidence interval of [0.084, 0.151], which does not contain 0, it indicates that there is a significant mediating effect of BA between EA and HPL, and therefore H4b is supported.

The standardized mediating effect of BA between SC and HPL is 0.119, with a confidence interval of [0.086, 0.158], which does not contain 0, it indicates that there is a significant mediating effect of BA between SC and HPL, and H4c is affirmed.

#### 3.1.6 Moderated mediation analysis

The moderating effect of SMU in the relationship between BA and HPL is tested. BA exerts a significant positive influence on HPL (*p* < 0.001, *β* = 0.379). SMU also has a significant positive effect on HPL (*p* < 0.05, *β* = 0.084). Moreover, the interaction term of SMU × BA shows a significant positive impact on HPL (*p* < 0.001, *β* = 0.147). Hence, SMU plays a positive moderating role between BA and HPL.

Table 5 indicates that the disparity in the mediating effect of BA between ER and HPL between high-level and low-level SMU is 0.084, with a confidence interval of [0.032, 0.136], which does not contain 0, Thus, it is regarded that SMU has a significant moderating effect on the mediation of BA between ER and HPL, H5a is validated.

**Table 5:** Result of moderated mediation model.

Mediation pathways	Conditional	Bootstrap β	S. E.	95%CI	
				Lower	Upper
ER → BA→HPL	Low (−SD)	0.063	0.015	0.034	0.095
	Median (Mean)	0.105	0.016	0.074	0.139
	High (+SD)	0.147	0.022	0.105	0.193
	Diff (High-Low)	0.084	0.027	0.032	0.136
EA → BA→HPL	Low (−SD)	0.070	0.016	0.040	0.104
	Median (Mean)	0.117	0.017	0.086	0.152
	High (+SD)	0.164	0.024	0.119	0.212
	Diff (High-Low)	0.094	0.029	0.037	0.151
SC → BA→HPL	Low (−SD)	0.072	0.018	0.039	0.109
	Median (Mean)	0.119	0.018	0.087	0.158
	High (+SD)	0.167	0.024	0.124	0.217
	Diff (High-Low)	0.095	0.030	0.036	0.154

The disparity in the mediating effect of BA between EA and HPL between high-level and low-level SMU is 0.094, with a confidence interval of [0.037, 0.151], which does not contain 0, Thus, it is regarded that SMU has a significant moderating effect on the mediation of BA between EA and HPL, H5b is established.

**Figure 2:**
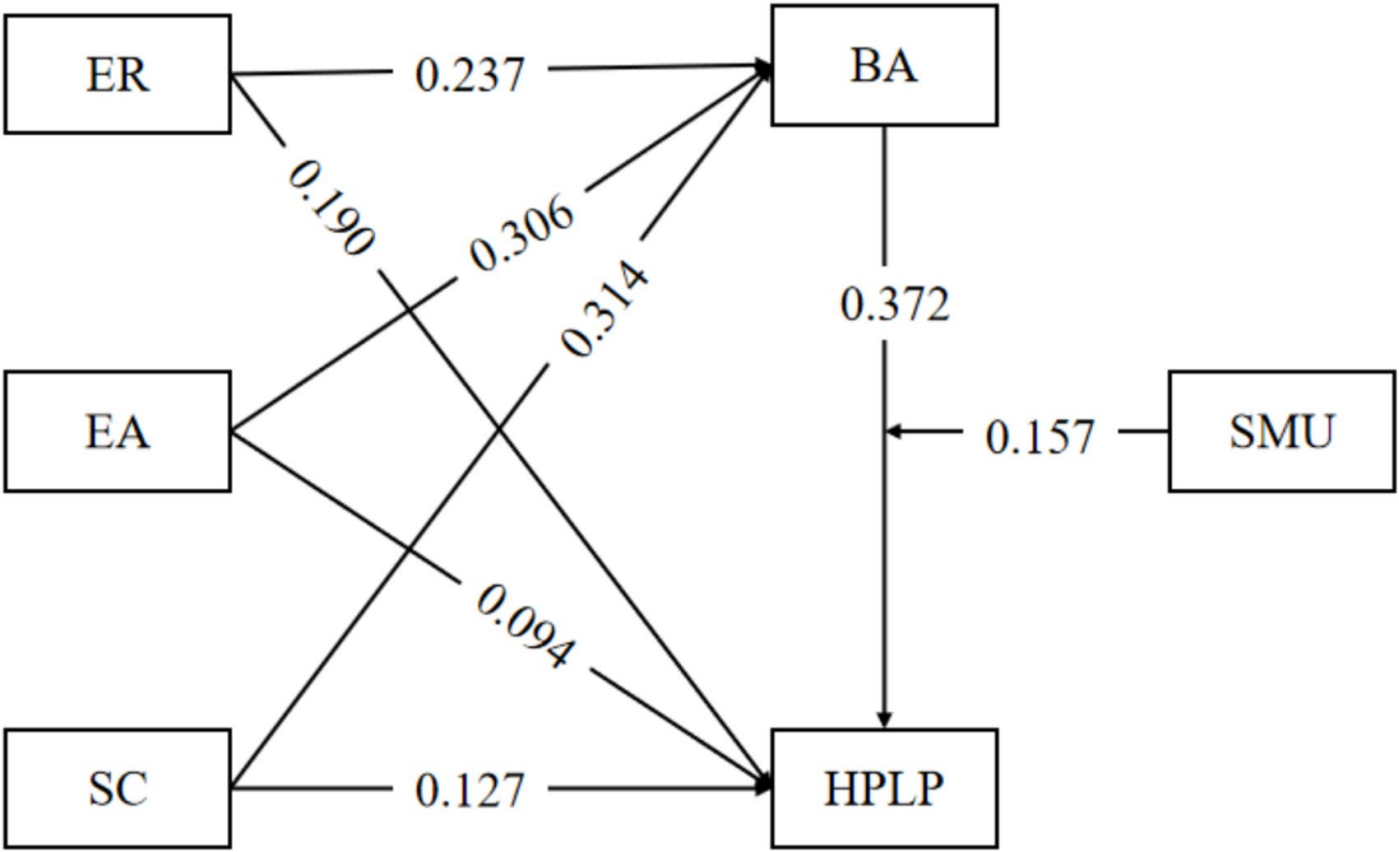
The path diagram of the SEM for the influencing factors of adolescents’ HPL.

The disparity in the mediating effect of BA between SC and HPL between high-level and low-level SMU is 0.095, with a confidence interval of [0.036, 0.154], which does not contain 0, Thus, it is regarded that SMU has a significant moderating effect on the mediation of BA between SC and HPL, H5c is affirmed. In this study, the relationships among all variables and their specific values are visually presented in Figure 2.

**Figure 3:**
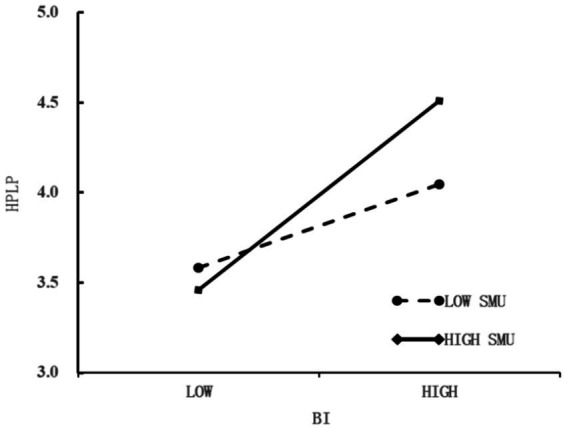
The simple slope indicating the moderation effects.

To present the moderating role of social media use on the relationship between body appreciation and health-promoting behaviors in a more intuitive manner, a diagram for decomposing the moderating effect is drawn, as depicted in Figure 3. The positive influence of body appreciation on health-promoting behaviors under high social media usage is greater than that under low social media usage. That is to say, as social media usage increases, the positive impact of body appreciation on health-promoting behaviors gradually strengthens. This demonstrates that social media use has a positive moderating effect on the relationship between body appreciation and health-promoting behaviors.

### 3.2 Fuzzy-set qualitative comparative analysis (fsQCA)

#### 3.2.1 Calibration

As presented in Table 6, in this study, the direct calibration method is employed. The 95th percentile, median, and 5th percentile of the sample descriptive statistics are, respectively, set as the anchors for full membership, the crossover point, and full non-membership. Additionally, to prevent cases from being eliminated due to the membership degree in the antecedent condition or result set being exactly 0.50, a constant of 0.001 is added to all conditions and results with a membership degree of 0.50.

**Table 6:** Data calibration.

Variable	Fully in (95%)	Crossover point (50%)	Fully out (5%)
Outcome variable			
HPL	4.000	2.993	2.128
Conditional variable			
ER	6.000	5.000	3.333
EA	6.000	5.000	3.000
SC	6.000	5.000	3.000
BA	5.000	4.000	2.300
SMU	5.000	3.333	1.333

#### 3.2.2 Analysis of necessary conditions

The necessity of individual conditions for HPL is examined. Table 7 reveals that the necessary consistency of individual conditions for HPL with respect to the outcome is all less than 0.9 (92). This indicates that ER, EA, SC, BA, and sSMU are none of the necessary conditions for high health-promoting behaviors.

**Table 7:** Necessary condition analysis.

Condition	High HPL	
	Consistency	Coverage
High ER	0.796	0.766
NOT High ER	0.511	0.540
High EA	0.792	0.747
Not High EA	0.513	0.555
High SC	0.814	0.749
Not High SC	0.502	0.558
High BA	0.862	0.788
Not High BA	0.456	0.511
High SMU	0.695	0.684
Not High SMU	0.616	0.635

#### 3.2.3 Sufficient configurations of conditions using fsQCA

In the configurational sufficiency analysis where high HPL serve as the outcome variable, the case frequency threshold is set at 1, and the original consistency threshold is set to 0.8. As indicated in Table 8, there exist three configurations capable of generating high health-promoting behaviors. The consistency of the overall solution is 0.875, and the coverage of the overall solution is 0.689. The conditional configurations derived from the analysis results can account for 68.9% of the cases. Hence, the analysis results obtained in this study possess a certain degree of explanatory power.

**Table 8:** Solutions regarding the presence and absence of high-perceived accessibility.

Configurations	High HPL			Not high HPL	
	S1	S2	S3	NS1	NS2
ER	**⬤**	**⬤**	**⬤**	⊗	⊗
EA		**⬤**			⊗
SC	**⬤**		**⬤**	⊗	
BA	**⬤**	**⬤**	**⬤**	⊗	⊗
SMU		**⬤**	**⬤**		**⬤**
Raw coverage	0.670	0.520	0.527	0.564	0.419
Unique coverage	0.186	0.036	0.043	0.186	0.040
Consistency	0.879	0.927	0.921	0.911	0.948
Solution consistency	0.749			0.604	
Solution coverage	0.873			0.911	

⬤ Indicates the presence of a core condition, ⊗ indicates the absence of a core condition.

S1: ER—SC—BA, this means that under the conditions of high ER, SC, and BA, even if SMU is not high, a result of HPL can be produced.

S2: ER—EA—BA—SMU, this means that under the conditions of high ER, EA, BA, and SMU, even if SC is not high, a result of high HPL can be produced.

S3: ER—SC—BA—SMU, this means that under the conditions of high ER, SC, BA, and SMU, even if EA is not high, a result of HPL can be produced.

The consistency of the overall solution for the configurational analysis of non-HPL is 0.911, and the coverage of the overall solution amounts to 0.604.

NS1 regards ER, SC, and BA as the core absent conditions. This implies that when ER, SC, and BA are all at a low level, the obtained HPL will not be high.

NS2 takes ER, EA, and BA as the core absent conditions and SMU as the core existing condition. This indicates that when ER, EA, and BA are all at a low level, even if SMU is present, the obtained HPL will not be high.

#### 3.2.4 Variation of consistency and calibration threshold

Upon changing the case frequency threshold to 2, the original consistency value and the PRI consistency threshold are in accordance with those in the hypothesis test. Moreover, the coverage and consistency of the overall solution remain unaltered, suggesting that the sufficiency analysis results of high health-promoting behaviors in this study are stable. When the PRI consistency is adjusted to 0.65, the original consistency value and the frequency threshold remain consistent with those in the hypothesis test. The coverage and consistency of the overall solution of the study do not change substantially, indicating that the sufficiency analysis results of high health-promoting behaviors in this study possess a certain degree of stability.

## 4 Discussion

The PLS-SEM results indicate a significant positive correlation between each constituent of emotional competence (emotional regulation, empathy, and self-control) and the healthy-promoting lifestyle of adolescents, thus supporting hypotheses H1a, H1b, and H1c. This result is in line with previous discoveries. Nables adolescents to actively modulate their emotions to influence behavior (93), thereby facilitating the adoption of better adaptive behaviors and even established behavior habits. In the post-pandemic era, adolescents who have personally endured the situation of regional lock downs have developed cognition regarding the effectiveness of a health-promoting lifestyle against large-scale public health threats. This experience has provided a real-world setting in which emotion regulation can be applied to cope with life challenges (94). Under such circumstances, when adolescents encounter behaviors, situations, and lifestyles that are detrimental to health in their daily lives, they can avoid making impulsive, emotionally driven decisions and gradually establish a health-promoting lifestyle. Adolescents with higher levels of EA can better able to understand their own emotions and feelings while sensing and experiencing the emotional states of others. A higher level of EA implies a greater degree of concern for personal health (95). When experiencing stress, anxiety, or fatigue, they will be more acutely aware of the impact of these negative emotions on the body and thus take positive countermeasures, such as resting, relaxing, or engaging in appropriate exercise to relieve stress and promote physical and mental well-being. Moreover, EA is intimately associated with prosocial behavior (96). Within social norms, HPL is often viewed as a behavioral pattern that conforms to social and personal lifestyle tendencies and is conducive to the overall development of individuals and society. For instance, maintaining personal health can reduce excessive utilization of medical resources and alleviate the social burden. This offers one plausible explanation for the relationship between empathy and health-promoting lifestyles among adolescents. Adolescents with strong SC are better equipped to forgo short-term gratifications in pursuit of long-term goals. In this study, those with higher SC are more likely to recognize the long-term consequences of unhealthy behaviors, such as smoking (48) and excessive alcohol (47) consumption. Such life habits that are harmful to the entire life cycle do not conform to long-term health interests, prompting adolescents to pay more attention to cultivating their own behavior patterns and living habits (45), resist harmful temptations, and naturally develop a health-promoting lifestyle.

Furthermore, the relationships between ER, EA, SC and BA were confirmed. Hypotheses H2a, H2b, and H2c are supported. The relationship between BA and HPL is affirmed, and H3 is verified. These findings reinforce the positive association between emotional competence and body appreciation, contrasting with prior studies that emphasized emotional dysregulation and distorted body image (73). Effective emotion regulation helps individuals maintain a positive emotional state and enhances self-efficacy (97). Such a person is more inclined to focus on their own merits and strengths, including various aspects of the body, and consequently raise the degree of appreciation for the body. The preventive function of emotional regulation in preventing body dissatisfaction has been confirmed (98). Emotional regulation strategies are also widely applied in the intervention processes of body shame and disorders of body appreciation (99).

This study also establishes a connection between EA and BA. A relatively high level of EA is significantly correlated with less negative perceptions of obese individuals, indicating a direct link between EA and BA. In clinical settings, overweight adolescents encounter the stereotypes of body image and the impediments of misaligned body appreciation daily. While previous studies have highlighted how empathy influences perceptions of others’ bodies and how received empathy affects one’s own body image, this study extends the literature by demonstrating that adolescents with higher empathy also report more positive appreciation of their own bodies. This can be explained by the capacity of empathy to promote acceptance of bodily diversity, leading to a more objective and inclusive self-body image and reducing the risk of low body appreciation. Similarly, adolescents with strong self-control tend to focus more on body functionality and health rather than appearance. They recognize that maintaining physical well-being holds greater long-term value than conforming to external aesthetic ideals. As a result, they evaluate their bodies more rationally and are less influenced by unrealistic social beauty standards. This health-oriented mindset translates into more positive body evaluation and higher levels of body appreciation. Body appreciation is closely associated not only with health-seeking behaviors—such as contraceptive use (100), and sun protection (101), etc., but also significantly correlated with health-promoting lifestyles [such as dietary patterns (69), avoiding excessive alcohol consumption and smoking (102), cancer screening (101), etc.]. This validates Ramseyer’s assertion that body appreciation can not only reduce unhealthy and dangerous behaviors but generate more protective actions (103). Therefore, efforts to enhance adolescents’ body appreciation should aim not only at reducing body dissatisfaction but also at strengthening the role of body appreciation as a psychological asset that supports mental health and motivates health-promoting behavior patterns.

The mediation analysis indicates that BA mediates the relationships between ER and HPL, EA and HPL, as well as SC and HPL. This suggests that the social emotional competence of adolescents (emotional regulation, empathy, and self-control) can not only directly enhance the HPL but also exert an influence on it via BA. This discovery offers a novel perspective and potential pathway for promoting the healthy lifestyle of adolescents. Specifically, we can foster the formation of a health-promoting lifestyle by cultivating adolescents’ social emotional competence and enhancing their levels of emotional regulation, empathy, and self-control, thereby strengthening their appreciation of their own bodies. Alternatively, interventions could focus directly on enhancing positive body image to facilitate the development of sustainable healthy lifestyles.

The moderation analysis reveals that SMU moderates the second stage of the mediated pathways through which BA links ER, EA, and SC to HPL. Hypotheses H5a, H5b, and H5c are thus supported. This result offers a nuanced viewpoints to earlier studies. In the past, research has consistently regarded social media use as a risk factor for body appreciation and health-promoting lifestyle (104, 105). Previous work suggested that frequent social media exposure—particularly to unrealistic beauty ideals—could undermine body appreciation (82) and subsequently weaken the positive impact of emotional regulation on health-promoting lifestyle (106). However, as social consciousness evolves, social media platforms have also become channels for disseminating diverse body-positive messages (72), as well as the recently advocated health-promoting lifestyle (71) on a global scale (including regular diet, fitness, meditation, etc.), has expanded its influence range through social media. As a result, in the case of high SMU by adolescents, the higher the degree of BA, the higher the level of HPL.

The fsQCA results reveal multiple configurations among ER, EA, SC, BA, and SMU that are associated with high levels of HPL for adolescents. Specifically, three distinct causal configurations were found to lead to this outcome. Configuration 1 reveals that in the absence of SMU and EA involvement, a high degree of HPL can also be generated through the synergistic effect of high ER, SC, and BA. This finding suggests that adolescents with superior emotional regulation ability and a higher level of BA are more inclined to actively understand their own bodies, be guided by continuous health benefits, and actively mobilize emotional resources for self-control (43), thereby establishing and maintaining a health-promoting lifestyle. Configuration 2 follows an ER-EA-BA-SMU pattern. It implies that under the circumstances of high ER, EA, BA, and SMU, even if SC is not particularly high, a result of high HPL can still be achieved. This underscores that the influence of emotional competencies is shaped by both body appreciation and social media contexts (82). This outcome suggests that the choice of a health-promoting lifestyle for adolescents is the consequence of the combined effect of adolescents’ own factors and external elements. Adolescents with stronger ER can sift through and distinguish different body image presentations and comments on body images under the social standard during SMU and generate EA. Thus, they can establish autonomous body image cognition and body appreciation and expedite the formation of a health-promoting lifestyle. Configuration 3 represents an ER-SC-BA-SMU pattern. It signifies that under circumstances of high emotional regulation, self-control, body appreciation, and social media utilization, irrespective of the level of adolescents’ empathy capacity, a health-promoting lifestyle will materialize. In addition, the fsQCA identified two configurations associated with the absence of a high HPL. The first configuration reveals that a non-high health-promoting lifestyle for adolescents is jointly brought about by low levels of emotional regulation, self-control, and body appreciation. The second configuration indicates that the absence of emotional regulation, empathy, and body appreciation will result in a non-high health-promoting lifestyle even when social media use is present. In addition, the results of fsQCA also discovered two causal models that lead to a non-high health-promoting lifestyle for adolescents. The first configuration shows that a non-high health-promoting lifestyle for adolescents is jointly caused by low levels of emotional regulation, self-control, and body appreciation. The second configuration indicates that the absence of emotional regulation, empathy, and body appreciation will result in a non-high health-promoting lifestyle even when social media use exists.

These findings underscore the relevance of emotional competence in shaping adolescents’ health-promoting lifestyles, aligning with previous empirical work. Notably, in all solutions for a high health-promoting lifestyle, at least one-dimensional structure of adolescent emotional competence is present. However, none of these components along constitutes a necessary condition. This suggests that the various components of adolescent emotional competence can achieve functional complementarity or mutual substitution to a certain extent.

## 5 Conclusion

This study illustrates how integrating PLS-SEM and fsQCA can function as an extended analytical approach to offer more comprehension for research and practice. By combining these methods, explore more deeply the influences of various components of emotional competence (emotional regulation, empathy, self-control) on adolescents’ health-promoting lifestyle, as well as the psychological mechanisms through which body appreciation and social media use affect emotional competence and adolescents’ health-promoting lifestyle. The results indicate that emotional regulation, empathy, and self-control can not only directly and significantly impact adolescents’ health-promoting lifestyle but also operate indirectly adolescents’ health-promoting lifestyle via body image. Furthermore, social media use plays a moderating role in this relationship. Emotional regulation, empathy, self-control, body image, and social media use can also act as independent antecedent conditions and have an impact on adolescents’ health-promoting lifestyle in different configurations. Collectively, these findings provide a novel perspective for in-depth exploration of the internal relationship and influence mechanism between adolescents’ emotional competence and behavior formation.

This study provides practical implications for families and schools to assist adolescents in establishing a health-promoting lifestyle. In terms of parenting, families should place greater emphasis on the development and changes of adolescents’ emotional competence and guide them to view body images on social media accurately, form a rational body image cognition, and establish a good level of body appreciation, thereby cultivating a health-promoting lifestyle. In school settings, educational appropriate aesthetic concepts and health awareness should be imparted to students in physical education and health courses, physiology and hygiene and other related courses. Educators should also remain attentive to students’ social media habits in order to strengthen the school’s role in fostering emotional competence, promoting the healthy and reasonable use of social media. This will help adolescents establish and maintain a health-promoting lifestyle and achieve the ideal goal of health throughout the life cycle through a health-promoting lifestyle.

Future studies can further investigate the specific mechanisms linking different dimensions of emotional competence to various aspects of health-promoting lifestyles, as well as the disparities in the impacts between different levels of body appreciation and the extent of adolescents’ health-promoting lifestyles. In addition, studies may explore how the cultivation of emotional competence can enhance the quality and sustainability of health-promoting behaviors, thereby informing more targeted and effective strategies to support adolescent health development.

## Data Availability

The raw data supporting the conclusions of this article will be made available by the authors, without undue reservation.
